# A Novel Method for Colorectal Cancer Screening Based on Circulating Tumor Cells and Machine Learning

**DOI:** 10.3390/e23101248

**Published:** 2021-09-25

**Authors:** Eleana Hatzidaki, Aggelos Iliopoulos, Ioannis Papasotiriou

**Affiliations:** 1 Research Genetic Cancer Centre SA (RGCC), 53100 Florina, Greece; hatzidaki.eleana@rgcc-genlab.com (E.H.); iliopoulos.aggelos@rgcc-genlab.com (A.I.); 2 Research Genetic Cancer Centre International GmbH, 6300 Zug, Switzerland

**Keywords:** colorectal cancer, circulating tumor cells, flow cytometry, machine learning, SVM, SMOTE

## Abstract

Colorectal cancer is one of the most common types of cancer, and it can have a high mortality rate if left untreated or undiagnosed. The fact that CRC becomes symptomatic at advanced stages highlights the importance of early screening. The reference screening method for CRC is colonoscopy, an invasive, time-consuming procedure that requires sedation or anesthesia and is recommended from a certain age and above. The aim of this study was to build a machine learning classifier that can distinguish cancer from non-cancer samples. For this, circulating tumor cells were enumerated using flow cytometry. Their numbers were used as a training set for building an optimized SVM classifier that was subsequently used on a blind set. The SVM classifier’s accuracy on the blind samples was found to be 90.0%, sensitivity was 80.0%, specificity was 100.0%, precision was 100.0% and AUC was 0.98. Finally, in order to test the generalizability of our method, we also compared the performances of different classifiers developed by various machine learning models, using over-sampling datasets generated by the SMOTE algorithm. The results showed that SVM achieved the best performances according to the validation accuracy metric. Overall, our results demonstrate that CTCs enumerated by flow cytometry can provide significant information, which can be used in machine learning algorithms to successfully discriminate between healthy and colorectal cancer patients. The clinical significance of this method could be the development of a simple, fast, non-invasive cancer screening tool based on blood CTC enumeration by flow cytometry and machine learning algorithms.

## 1. Introduction

Cancer is extremely complex and heterogeneous. It includes various processes (e.g., evading growth suppressors, resisting cell death, replicative immortality), which manifest as cancer’s irregular dynamics in multi-level spatio-temporal scales. In particular, at the molecular level, a large number of interacting molecules (proteins, lipids and ions) constitute a complex network, which results in complex intracellular signaling, non-linear reaction kinetics, gene mutations and dysregulations, regulatory circuits, pathway cross-talks and others [1,2,3,4]. The processes are non-linear, and the formation of the hierarchies themselves may be discontinuous [5]. In addition, cancer has many (more than 100 distinct types) incarnations. Different categories of cancer exist such as carcinoma, sarcoma, leukemia, lymphoma and myeloma and central nervous system cancers, all depending on where carcinogenesis was initiated (e.g., skin, bone, blood, brain), as well as different types such as bladder, breast, colon and rectal, endometrial, lung, pancreatic, prostate, etc. All these types and/or categories are characterized by unique features and growth dynamics, increasing the already high levels of complexity needed to be confronted by scientists for dealing with carcinogenesis in prevention, early detection, treatment management and screening post-treatment.

Colorectal cancer (CRC) is one of the most common types of cancer, and it can have a high mortality rate if left untreated or undiagnosed. It is estimated that 10% of all annually diagnosed cancers are CRC [6]. Risk factors include both environmental and genetic ones. Apart from age, obesity, lack of exercise, smoking, alcohol consumption and dietary habits are also implicated [7]. Family history can be a contributing factor in 10–20% of CRC cases [8]. Hereditary CRC syndromes include polyps, Lynch syndrome, inflammatory bowel syndrome and type 2 diabetes. Colon cancer survival rates are poor if diagnosed late. Furthermore, the disease becomes symptomatic mainly at advanced stages. This highlights the importance of early screening. At present, guidelines do not recommend screening for CRC at ages lower than 50, unless there is a family history [9]. However, the fact that CRC incidences show an increase in younger age groups has prompted the re-consideration of the guidelines. Colonoscopy is the current reference method for CRC screening. It is an invasive procedure and requires the use of sedation or anesthesia. CRC biomarkers detected in blood could be an attractive alternative. Although a few prognostic biomarkers are known, such as carcinoembryonic antigen (CEA), microsatellite instability (MSI) and BRAF mutation, there are no diagnostic biomarkers available in clinical practice.

Despite the significant advances in the field, colorectal cancer therapy, recurrence and metastasis continue to face difficulties and new challenges due to cancer’s inherent complexity. Therefore, detailed biological information (e.g., differences between cancer states and healthy states and/or between cancer subtypes) and the utilization of advanced mathematical methods are of great importance [10,11,12,13,14]. Specifically, in the last two decades there has been an exponential growth of Machine Learning (ML) algorithms utilized for addressing difficult healthcare challenges including complex biological abnormalities such as cancer [15,16,17,18,19]. ML has introduced novel biomarkers for cancer diagnosis, designed novel personalized drugs and delivered potential treatment strategies [20,21,22,23]. For achieving these targets, scientists analyze various types of input data [15], such as genomic (SNPs, mutations, microarrays), proteomic (specific protein biomarkers, 2D gel data, mass spectral analyses), clinical (histology, tumor staging, tumor size, age, weight, risk behavior, etc.), high-resolution images (which are involved almost in every cancer diagnosis), demographic, epidemiological or combinations of some of these. The analyses were based on a variety of ML techniques utilized for the development of predictive models. These include Artificial Neural Networks (ANNs), Deep Learning (DL), Bayesian Networks (Bns), Support Vector Machines (SVMs), Decision Trees (Dts) and others [16].

In this direction, [24] developed a deep learning-based method to measure the similarity between CRC tumors and cancer cell lines. The datasets considered contained copy number alterations, gene expression and point mutations. The model learns latent factors that represent clinically relevant patterns and explains the variability of molecular profiles across tumors and cell lines, providing best-matching cell lines to different cancer subtypes. In addition, [25] developed a multi-parameterized ANN to score the risk of colorectal cancer based solely on personal health data from the National Health Interview Survey (NHIS). The ANN was tested per Transparent Reporting of Multivariable Prediction Model for Individual Prognosis or Diagnosis (TRIPOD) level 2a and level 2b protocols. The result showed that the ANN is comparable to current methods of scoring CRC risk (including those using biomarkers). Another example is given by [26]. In this comparative study, seven ML algorithms were evaluated in combination with six imputation methods for missing data, all trained and cross-tested with the NHIS and PLCO datasets concerning CRC. The optimal model was an ANN with Gaussian expectation-maximization imputation, which can be used as a non-invasive and cost-effective tool to screen the CRC risk in large populations effectively using only personal health data. Finally, a state-of-the-art transfer-learned deep convolutional neural network was developed recently by [27], who proposed a novel patch aggregation strategy for clinic CRC diagnosis using weakly labeled pathological whole-slide image (WSI) patches. This approach yielded promising results, often even better than most of the experienced expert pathologists when tested in diagnosing CRC. For more information on CRC screening, diagnosis and treatment based on other AI applications, the interested reader can refer to a very thorough review by [28].

In this study, we built an ML classifier for discriminating colorectal cancer samples from non-cancer, healthy samples. The ML classifier was based on Support Vector Machines (SVM), which were chosen as an appropriate approach for classifying colorectal cancer and non-cancer/healthy samples. We used SVM since they are among the most commonly applied ML algorithms within the field of cancer research and more generally in computational biology [29,30,31,32,33], exhibiting accurate predictive performance. SVM can be used to overcome classification problems concerning datasets with small sample size, high dimensionality and nonlinearity with good generalization capability. In this direction, the SVM classifier was also compared with other classifiers, developed by methods frequently utilized in ML applications, using larger over-sampling datasets generated by the SMOTE algorithm [34]. 

As a dataset, we used experimental data derived from circulating tumor cells (CTCs) [35] detected by flow cytometry, which can be promising prognostic biomarkers in CRC [36]. In particular, circulating CTCs are cancer cells that are shed from the tumor and travel in blood circulation. CTCs actively leave the tumor tissue and invade the blood stream using a process known as epithelial-to-mesenchymal transition (EMT). During EMT, cancer cells lose their epithelial characteristics and acquire mesenchymal ones. This allows them to become mobile and migrate from the primary to the metastatic site [37]. Today, there is only one FDA-approved detection technique for CTCs. It relies on EpCam-positive and CD45-negative immunoselection of fixed cells. CTCs are then detected using high-resolution imaging combined with immunocytofluorescent staining [38]. The system therefore detects CTCs by counting cells positive for fluorescent signal co-localization in an image captured by a camera. However, EpCam, being an epithelial marker, limits the ability to evaluate CTCs from tumors that have no EpCam expression, or cancer cells that have undergone EMT [39]. More importantly, today’s technologies for CTC determination rely mainly on traditional microscopy imaging and therefore suffer from the same limitations. Well focused images are imperative for image analysis; ideally, images should be viewed under different light sources, phase contrast, bright-field and fluorescence, and finally, there is a limitation to the pixel information a microscope can deliver [40]. On the other hand, flow cytometry is a powerful and sensitive cell analysis technique that detects fluorescent signals as cells pass one by one in front of a light source. If the cytometer is a sorter, cells can also be isolated alive and cultured for downstream analyses. We have developed a method for CTC determination in whole blood using flow cytometry. CTCs were defined as CD45-negative, CD31-negative and pan-cytokeratin-positive cells in peripheral blood cells. It was found that our method of CTC detection by flow cytometry had a sensitivity of 86.2% and specificity of 83.9% [41].

The aim of this study was, firstly, to validate our method for CTC determination, and secondly, to use these data to perform binary classification between colorectal cancer and healthy samples. The clinical significance of this method could be the development of a non-invasive cancer screening tool based on blood CTC enumeration by flow cytometry and ML.

## 2. Materials and Methods

### 2.1. Sample Collection

This study was not a clinical trial and did not include any interventions. The study was reviewed and approved by our institutional ethics committee. Informed consent was obtained from all patients. Blood samples from a total of 41 healthy individuals/non-cancer patients and 41 CRC patients were collected in sterilized 50-mL falcon tubes containing 7 mL 0.02 M EDTA as an anti-coagulant. Healthy individuals were identified as healthy/non-cancer by their physicians.

### 2.2. Sample Preparation

A total of 2 mL of blood was mixed with 2 mL of fetal bovine serum in 15-mL centrifuge tubes to regain the cells’ shape. The samples were then centrifuged at 1200 rpm for 10 min at room temperature and the supernatant was discarded. A total of 100 μL of sample was transferred to round-bottom tubes for flow cytometry analysis.

### 2.3. Antibodies and Staining Procedure

Antibodies used were CD45-PE/Cy7, CD31-RPE and pan CK-PE/Cy5. Samples were fixed and permeabilized using LEUCOPERM according to the manufacturer’s instructions. Briefly, first samples were stained with surface antibodies for 20 min (CD45 and CD31, 5 μg/mL each), washed with PBS and then fixed with 100 μL Leucoperm Reagent A, washed with PBS, permeabilized with 100 μL Reagent B and stained intracellularly with 5 μg/mL pan-CK antibodies for 20 min and washed again with PBS. After the last wash, cells were re-suspended in 500 μL PBS and were ready for acquisition in a Beckman Coulter FC500 cytometer.

### 2.4. Sample Blinding

A total of 31 healthy and 31 cancer samples were known to the investigators and were used for the training and validation of the algorithm. Twenty samples were blinded by using 5-digit codes and were used for prediction (test) analysis.

### 2.5. Sample Acquisition and FCS Data Analysis

**Figure 1:**
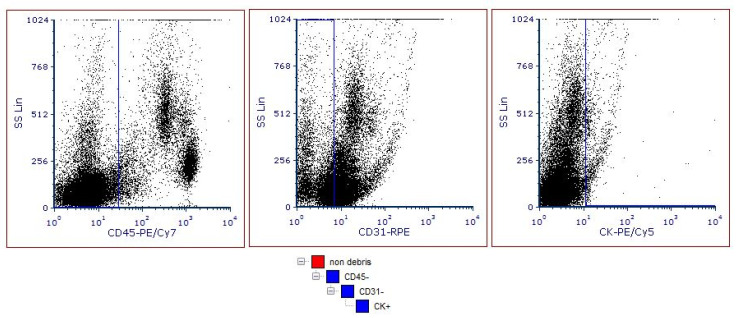
Gating strategy for the identification of CTCs in PBMCs. First on the left plot shows exclusion of CD45-positive cells (hematopoietic); second plot shows exclusion of CD31-positive cells (epithelial); third plot shows selection of pan-CK-positive cells.

Circulating tumor cells were defined as CD45-negative, CD31-negative and pan-cytokeratin-positive cell populations. Non-hematological cells were gated out using a CD45-negative selection. The endothelial cells were then removed using a CD31-negative gating selection. Tumor cells were identified by pan-CK-positive selection. Unstained samples were used as a negative control for gating. FCS Express software was used for fcs data analysis. Figure 1 shows the gating strategy in FCS Express, where the CD31-negative gate is set as a CD45-negative sub-gate, and the pan-CK-positive gate is set as a CD31-negative sub-gate.

### 2.6. Mathematical Analysis

#### 2.6.1. Two-Sample Kolmogorov–Smirnov Test

The two-sample Kolmogorov–Smirnov test [42] is a nonparametric hypothesis test that evaluates the difference between the Cumulative Distribution Functions (CDFs) of the two samples over the range of x in each dataset. The two-sided test uses the maximum absolute difference between the CDFs of the distributions of the two samples. The test statistic is:

(1) $$D^{*}=\underset{*}{max}\left(\left|{\hat{F}}_{1}\left(x\right)-{\hat{F}}_{2}\left(x\right)\right|\right),$$

where ${\hat{F}}_{1}\left(x\right)$ is the proportion of $x_{1}$ values less than or equal to x, and ${\hat{F}}_{2}\left(x\right)$ is the proportion of $x_{2}$ values less than or equal to x.

#### 2.6.2. Wilcoxon Rank Sum Test

The Wilcoxon rank sum test is a nonparametric test for two populations [43]. In particular, this test examines the null hypothesis that two samples are drawn from continuous distributions with equal medians, against the alternative hypothesis that they are not. The test assumes that the two samples are independent. The Wilcoxon rank sum test is equivalent to the Mann–Whitney U-test, which is a nonparametric test for equality of population medians of two independent samples X and Y. Specifically, the Mann–Whitney U-test statistic, U, is the number of times a y precedes an x in an ordered arrangement of the elements in the two independent samples X and Y. It is related to the Wilcoxon rank sum statistic in the following way: If X is a sample of size $n_{x}$, then:

(2) $$U=W-\frac{n_{x}\left(n_{x}+1\right)}{2}$$

#### 2.6.3. Support Vector Machines

In this paper, in order to solve this binary classification problem, we apply a powerful classifier, the support vector machine (SVM). SVM aims to create a decision boundary between two classes in order to predict the labels from one or more feature vectors [44,45]. This decision boundary is known as the hyperplane. Its orientation is crucial for the best separation of the closest data points from each of the classes. These closest points are called support vectors. In particular, for given a labeled training dataset:

(3) $$\left(x_{1},y_{1}\right),\ldots ,\left(x_{n},y_{n}\right),x_{i}\in R^{d}\wedge y_{i}\in \left(-1,1\right),$$

where $x_{i}$ is a feature vector representation and $y_{i}$ is the class label (negative or positive) of a training compound *i*, and the optimal hyperplane can be defined as:

(4) $$wx^{T}+b=0,$$

where w is the weight vector, x is the input feature vector and b is the bias. In the best case scenario, w and b would satisfy the following inequalities for all elements of the training set:

(5) $$wx_{i}^{T}+b\geq 1\;if\;y_{i}=1,$$

(6) $$wx_{i}^{T}+b\leq -1\;if\;y_{i}=-1.$$

Therefore, the objective of training an SVM model is to find the proper w and b so that the hyperplane separates the data and maximizes the margin $1/{\left\|w\right\|}^{2}$.

However, many binary classification problems do not have a simple hyperplane as a useful separating criterion. For such problems, instead of using a linear SVM classifier, we can alternatively use the kernel method. This method enables us to model higher dimensional, non-linear models, while retaining nearly all the simplicity of an SVM separating hyperplane. Specifically, the kernel method transforms the data into higher dimensional spaces to make the data separable.

In general, a kernel function is defined as:

(7) G(x,y)=<f(x),f(y)>,

where *G* is the kernel function, *x* and *y* are *n* dimensional inputs and *f* is used to map the input from *n* dimensional to m dimensional space. Finally, the term <x,y> denotes the dot product. This class of functions includes polynomials and Radial Basis Function (RBF). In particular, polynomials (e.g., linear, quadratic, cubic) are defined as:

(8) $$G\left(x,y\right)={\left(1+x^{\prime }y\right)}^{p},$$

where *p* is some positive integer, while RBF kernel is defined as:

(9) $$G\left(x,y\right)=exp\left(-\|x-{y\|}^{2}\right).$$

Of course, the choice of kernel function, among other parameters, can greatly influence the performance (e.g., reduce or increase the classification probability error) of an SVM model. One can choose between the available kernels through trials and, depending on the nature of the problem, select the best one. One way to find the optimal kernel in a statistically rigorous fashion is by using cross-validation.

Particularly, cross-validation is a procedure used to avoid under- and overfitting [46]. It is a process in which the dataset is randomly partitioned into a training and a test set. In this paper, we used a *k*-fold cross validation procedure. In particular, this method splits the data randomly into *k* equal (or almost equal) parts. Then, the algorithm runs *k* times, using *k*-1 of the parts as a training set and the remaining part as a test set. Each time the algorithm runs, a different test set is used, so that over the *k* runs of the algorithm, all the instances in the dataset are used as a test set. The success of the algorithm is the sum of the correct classification over each of the runs. However, even cross-validation can overestimate the prospective performance of ML methods. Therefore, we also conducted a truly blind test in order to demonstrate the prospective capabilities of our cross-validated model [20].

#### 2.6.4. Comparison between Different Classifiers

One drawback of this study is the relatively small dataset, which can lead to biased models that are not generalizable. Therefore, in order to further test the generalizability of our method, we also compared the performances of many classifiers, in addition to the SVM classifier, developed by methods frequently utilized in ML applications. In particular, we developed optimizable models from classification trees [47], discriminant analysis [48], logistic regression [49], naïve Bayes [50], k-nearest neighbor (kNN) [51] and ensemble methods, including boosted trees, bagged trees (random forest), subspace discriminant, subspace kNN and RUSBoosted trees [50,51,52,53]. The hyperparameter search range for the different classifiers was: (a) Classification trees: *Maximum number of splits*: 1–163, *Split criterion*: Gini’s diversity index, Maximum deviance reduction. (b) Discriminant Analysis: Linear, Quadratic, Diagonal Linear, Diagonal Quadratic. (c) Naïve Bayes: *Distribution names*: Gaussian, Kernel, *Kernel type*: Gaussian, Box, Epanechnikov, Triangle. (d) SVM: *Multiclass method*: One-vs.-All, One-vs.-One, *Box constraint level*: 0.001–1000, *Kernel scale*: 0.001–1000, *Kernel function*: Gaussian, Linear, Quadratic, Cubic, *Standardize data*: true, false. (e) kNN: *Number of neighbors*: 1–82, *Distance metric*: City block, Chebyshev, Correlation, Cosine, Euclidean, Hamming, Jaccard, Mahalanobis, Minkowski (cubic), Spearman, Distance weight: Equal, Inverse, Squared inverse, *Standardize data*: true, false. (f) Ensemble: *Method*: Bag, GentleBoost, LogitBoost, AdaBoost, RUSBoost, *Number of learners*: 10–500, *Learning rate*: 0.001–1, *Maximum number of splits*: 1–163, *Number of predictors to sample*: 1–2.

In order to perform the comparison, since the dataset is small, we updated the original dataset, generating over-sampling datasets. Therefore, we tested the performance of all the above classifiers using the over-sampling datasets. In order to create the over-sampling datasets, we used a robust method named Synthetic Minority Over-sampling Technique (SMOTE) [34,54]. This is an over-sampling approach that creates synthetic minority class samples. This technique is widely used and performs better than simple over-sampling. In particular, the SMOTE samples are linear combinations of two similar samples from the minority class (x and $x^{R}$) and are defined as:

(10) $$s=x+u*\left(x^{R}-x\right),$$

where u is randomly chosen from U(0, 1) and differs for each SMOTE sample. This guarantees that a SMOTE sample lies on the line joining the two original samples used to generate it [34,54]. For more information on SMOTE and its updates, the interested reader can refer to [55].

#### 2.6.5. Performance Measures for Binary Classifiers

The performance analysis of the model can be measured in terms of sensitivity, specificity, accuracy and area under the curve (AUC). They are all based on true positives (TP, correctly predicted positive (cancer) samples); true negatives (TN, correctly predicted negative (non-cancer/healthy) samples), false positives (FP, normal samples wrongly predicted as being cancer samples) and false negatives (FN, cancer samples wrongly predicted as non-cancer/healthy) [56].

In particular, Accuracy is the percentage of correctly predicted samples, and is defined as:

(11) $$Accuracy=\frac{TP+TN}{TP+TN+FP+FN}\times 100\%,$$

and is used for estimating the overall performance of the classifier.

Sensitivity or True Positive Rate (TPR) is the percentage of samples correctly predicted as cancer samples, and is defined as:

(12) $$Sensitivity=\frac{TP}{TP+FN}\times 100\%.$$

The opposite of sensitivity is called False Negative Rate (FNR) or Miss Rate and is equal to FNR = 1 − TPR.

Specificity or True Negative Rate (TNR) is the percentage of samples correctly predicted as non-cancer/healthy samples, and is defined as:

(13) $$Specificity=\frac{TN}{TN+FP}\times 100\%.$$

Precision or Positive Predictive Value (PPV) is the percentage of samples correctly predicted as cancer from all positive predictions, and is defined as:

(14) $$Precision=\frac{TP}{TP+FP}\times 100\%$$

The opposite of Precision is False Discovery Rate (FDR) equal to FDR = 1 − PPV.

Area under the Curve (AUC) is a measure of the model’s overall performance. AUC for binary classification [56] is given by:

(15) $$AUC=\frac{1}{2}\left(\frac{TP}{TP+FN}+\frac{TN}{TN+FP}\right)$$

The maximum AUC is 1, which corresponds to a perfect classifier, while for a classifier that randomly assigns observations to classes, AUC = 0.5. Larger AUC values indicate better classifier performance. A rough rule of thumb is that the accuracy of tests with AUCs between 0.50 and 0.70 is low; between 0.70 and 0.90, the accuracy is moderate; and it is high for AUCs over 0.90 [57].

AUC is the primary statistic we obtain from a Receiver Operating Characteristics (ROC) curve [58], which plots the tradeoffs between sensitivity and 1-specificity. In particular, ROC graphs are two-dimensional graphs in which sensitivity (TPR) is plotted on the Y-axis and FPR (1-TNR) on the X-axis, for different thresholds of the classifier output. They are useful for organizing classifiers and visualizing their performance. In such a graph, the point (0, 1) represents perfect classification.

## 3. Results

**Figure 2:**
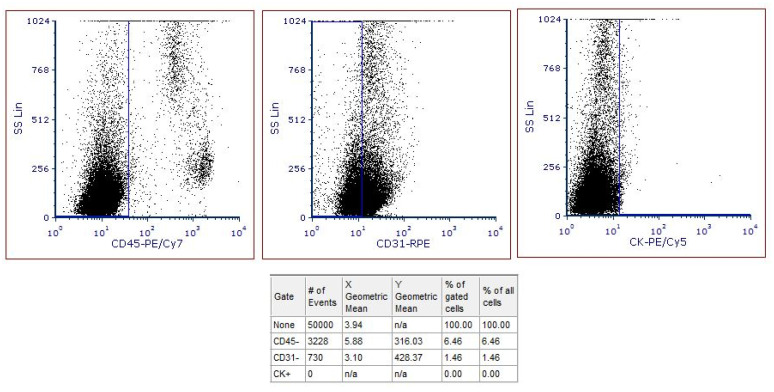
Representative analysis of a healthy sample. No cells were found to be CD45-/CD31-/CK+ as denoted in the column # of Events (number of Events).

**Figure 3:**
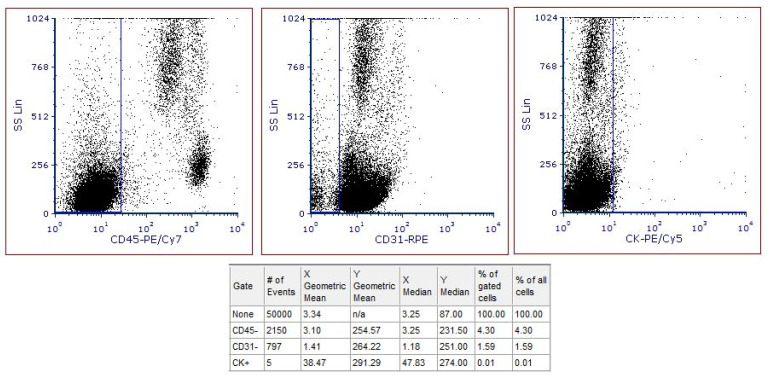
Representative analysis of a cancer patient sample. Five cells were found to be CD45-/CD31-/CK+ as denoted in the column # of Events (number of Events).

After data acquisition, CTCs from the 31 cancer and 31 healthy samples were calculated using FCS Express. Figure 2 shows a healthy sample analysis. No CTCs were found as it is denoted by the column # of Events (number of Events). Figure 3 shows a cancer sample. Five CTCs were found using the same method.

### 3.1. Statistical Tests

Before we built the classifier, we tested for differences between the cancer and non-cancer/healthy distributions and their medians. This was achieved using two non-parametric hypothesis tests, namely the two-sample Kolmogorov–Smirnov (KS) test and the Wilcoxon rank sum (WRS) test. Both tests revealed significant statistical differences in terms of distributions and medians. In particular, the KS test rejected the null hypothesis, namely that the data are from the same continuous distribution with a *p*-value equal to $1.85^{-6}\ll 0.05$. In addition, the WRS test rejected the null hypothesis, namely that the data are samples from continuous distributions with equal medians with a *p*-value equal to $2.86^{-7}\ll 0.05\;$. Therefore, the cancer and non-cancer/healthy samples have significant statistical differences, both in terms of their distributions as well as their medians. This information indicates that an efficient classifier can be built based on this dataset. All computations for the statistical tests were performed in MATLAB [59], using the Statistics and Machine Learning Toolbox.

### 3.2. SVM Classifier

We used a 5-fold cross validation and MATLAB’s Bayesian Optimization function *bayesopt* to find the best (optimized) classification SVM model. In particular, the hyperparameter search range included *box constraint level*: 0.001–1000, *kernel_scale*: 0.001–1000 and *kernel_function*: Gaussian, linear, quadratic, cubic. The optimized SVM model consisted of a quadratic kernel function (*scale* = 1, *order* = 3) and *box constraint level* equal to 3.0685. The data were standardized.

**Figure 4:**
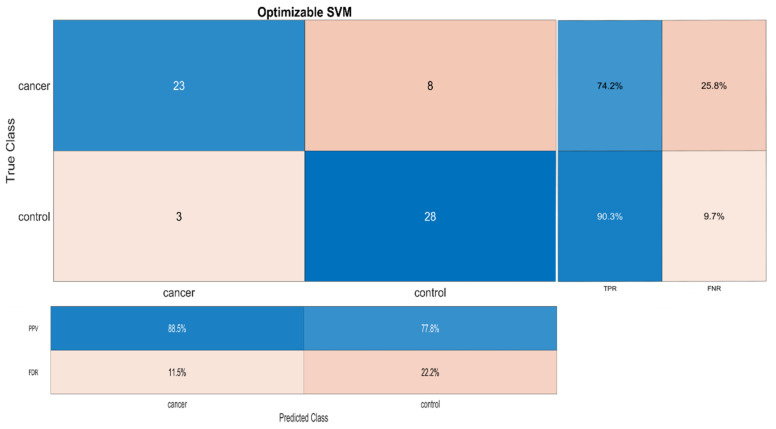
Confusion matrix for the optimized SVM classifier. In the big panel, the rows correspond to the true class, and the columns correspond to the predicted class. Diagonal and off-diagonal cells correspond to correctly and incorrectly classified observations, respectively. The sensitivity (TPR) is shown in the right panel, first column and miss rate (FNR) in the right panel, second column. Additionally, the precision (PPV) is shown in bottom panel, first row and false discovery rate (FDR) in the bottom panel, second row.

The results of the optimized SVM are shown in the confusion matrix (Figure 4). In particular, in this figure the total number of observations in each cell is presented (central panel). The rows correspond to the true class, and the columns correspond to the predicted class. Diagonal and off-diagonal cells correspond to correctly and incorrectly classified observations, respectively. As it can be seen in this panel, considering the cancer samples as positives, the true positives (TP) were found equal to 23, true negatives (TN) = 28, false positives (FP) = 3 and false negatives (FN) = 8. Based on these values, we estimated the performance measures using Equations (11)–(14). In particular, the accuracy of the classifier was found to be 51/62 × 100% = 82.3%.

In addition, in the right panel, the row summary displays the percentages of correctly and incorrectly classified observations for each true class. This panel shows that the sensitivity (TPR) is equal to 23/31 × 100% = 74.2% and the miss rate (FNR) is equal to 8/31 × 100% = 25.8%. This means that 23 samples were correctly classified as cancer samples and eight samples were falsely classified as non-cancer/healthy (false negatives) out of 31 cancer samples. Similarly, the specificity (TNR) is 28/31 × 100% = 90.3%, while 3/31 × 100% = 9.7% were falsely classified as cancer samples.

Finally, the bottom panel displays a summary of the percentages of correctly and incorrectly classified observations for each predicted class. Specifically, this panel shows the results concerning the precision (PPV) and False Discovery Rate (FDR) of the optimized SVM model. As it is shown, PPV is equal to 23/26 × 100% = 88.5% for the cancer samples and 28/36 × 100% = 77.8% for the non-cancer/healthy samples. The FDR is 100% − 88.5% = 11.5% for the cancer samples and 100% − 77.8% = 22.2% for the non-cancer/healthy samples, respectively.

**Figure 5:**
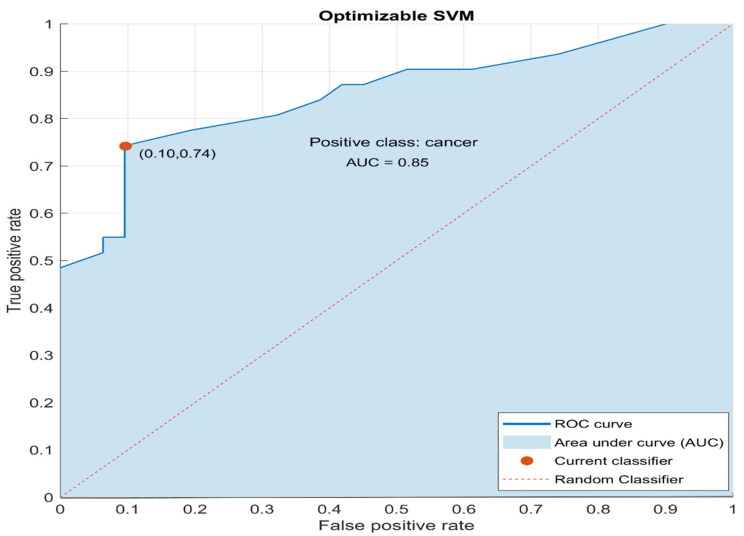
Receiver operating characteristic (ROC) curve for the optimized SVM classifier. The AUC is equal to 0.85. The optimal operation point for the current classifier is also shown (orange dot). The ROC curve of a random classifier is also shown (dotted red line).

In Figure 5, the ROC curve for the optimized SVM is shown. In the same figure, the AUC, the optimal point for the current classifier (orange dot) and the ROC curve for a random classifier (diagonal red dotted line) are also shown. The random classifier identifies an equal amount of positives and negatives correctly. Therefore, the AUC for a random classifier is 0.5. Any classifier that appears in the lower right triangle performs worse than random guessing. As it can be seen, in Figure 5, the AUC of the optimized classifier is 0.85 >> 0.5, indicating a moderate-to-high accuracy classifier [57].

In addition, as it is shown, the optimal point (the point that will result in the lowest number of overall errors: FN + FP) for the classifier is found for TPR = 0.74 and FPR = 0.10, near the Y-axis. Classifiers appearing on the left-hand side of an ROC graph are rather “conservative”, namely they make positive classifications only with strong evidence, making few false positive errors [58].

### 3.3. Blind Set

**Figure 6:**
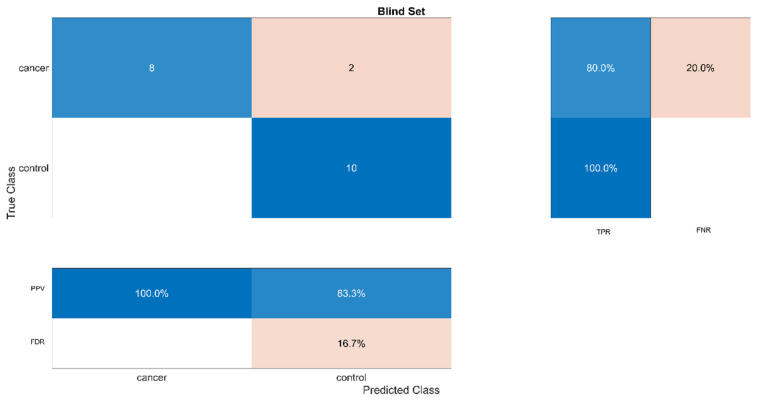
Similar to Figure 5. Confusion matrix of the optimized classifier for the blind set.

In order to further test the performance of the optimized SVM classifier, we examined its performance in a totally blind set. As mentioned, this set includes 10 cancer and 10 non-cancer/healthy samples. The results are summarized in Figure 6 and reveal that TP = 8, TN = 10, FP = 0 and FN = 2. Therefore, the accuracy in the blind set was found to be 18/20 × 100% = 90.0%. Moreover, the sensitivity (TPR) was found equal to 80.0%, the miss rate equal to 20.0%, the specificity equal to 100% and the precision equal to 100.0%. Finally, the AUC for the blind set was found equal to 0.98. All computations were performed in MATLAB [59] using the Statistics and Machine Learning Toolbox.

Overall, the results demonstrate that the SVM classifier, based on CTCs enumerated by flow cytometry, can successfully discriminate between healthy and colorectal cancer patients with high values of performance measures.

### 3.4. Comparison between Different Classifiers

In order to further test the generality of our results, we compared different classifiers developed by models frequently utilized in ML applications. All computations were performed in MATLAB [59] using the Statistics and Machine Learning Toolbox. The classifiers were developed on SMOTE-generated over-sampling datasets, using the MATLAB package *smote* [60]. This function synthesizes new observations based on existing (input) data and a K-nearest neighbor approach.

In addition, for the generation of the over-sampling datasets, both the training as well as the blind sets were taken into consideration. In particular, we generated six new datasets by varying both the amount of over-sampling (N) as well as the number of considered nearest neighbors (K). We considered N = 1 (2-fold observations), N = 3 (3-fold observations) and N = 10 (10-fold observations), and K = 5, 10, 20, 30. Therefore, since the initial set (including both the training and blind sets) consists of 41 colon cancer samples and 41 healthy samples, the resulting datasets contained: D1(N = 1, K = 5) = 164 samples, D2(N = 1, K = 10) = 164 samples, D3(N = 3, K = 10) = 328 samples, D4(N = 3, K = 20) = 328 samples, D5(N = 10, K = 20) = 902 samples and D6(N = 10, K = 30) = 902 samples.

The results of the performances of the classifiers are shown in Table 1 and Table 2. Specifically, in Table 1 we show the validation accuracy of the optimized models (the optimization of the models was performed using a 5-fold cross validation and MATLAB’s Bayesian Optimization function *bayesopt*). As it can be seen, all models achieved high validation accuracies, above 84%, while the differences are not so big, even for the linear benchmark method of logistic regression. This is expected, since we only have one feature as an input. The highest validation accuracies (%) were found for the classifiers based on SVM (D1—86.0, D2—89.0, D3—89.6, D4—87.2), for on ensemble classifiers (D1—86.0, D4—87.2, D6—88.2) and for on classification trees (D1—86.0, D5—87.6). 

We also estimated the AUC for each of the optimized models and the results are presented in Table 2. As it can be seen, all models achieved high values of AUC (≥0.84). In this case, the highest values were achieved by ensemble methods and in particular by Gentle Adaptive Boosting (GentleBoost) [61] for four datasets (D1—0.89, D2—0.89, D4—0.92, D5—0.94), and the Bootstrap Aggregation and Random Forest (Bag) [62] for one dataset (D3—0.92). Logistic regression also yielded the highest AUC for four datasets (D1—0.89, D4—0.92, D5—0.94, D6—0.95), whereas the other classifiers attained the highest performances for fewer datasets.

Taking into account the results concerning validation accuracies as well as AUC, it can be concluded that, even though all ML classifiers yielded high performances, SVM performed better according to the validation accuracy metric, while ensemble methods performed better according to the AUC metric. However, compared to AUC, accuracy is simpler and easier to interpret, while it is mostly used for evaluating supervised binary classifiers with balanced classes, taking into account both true positive as well as true negative predictions. Therefore, based on accuracy results, in this study, we chose SVM for developing efficient and robust ML classifiers.

## 4. Discussion

In the present study, we developed an SVM classifier for performing binary classification between colorectal cancer and non-cancer/healthy samples. The main feature used for the classification is the number of CTCs from cancer and non-cancer/healthy samples, as obtained from flow cytometry. In this study, 31 colorectal cancer and 31 non-cancer/healthy samples were used for the development of the SVM classifier. In addition, the SVM classifier was tested in a blind test set, which included 10 cancer samples and 10 non-cancer/healthy samples. Finally, in order to further test the efficiency and generalizability of the proposed method, we generated various over-sampling datasets by applying the SMOTE algorithm and used these datasets in order to develop and compare various ML classifiers.

The results of this study revealed the efficiency of the developed SVM classifier both on the training set as well as on the blind set. In particular, for the training set, the performance measures of the SVM classifier were found to be: accuracy equal to 82.3%, sensitivity (TPR) equal to 74.2%, miss rate (FNR) equal to 25.8%, specificity (TNR) equal to 90.3%, precision (PPV) equal to 88.5% and AUC equal to 0.85. For the blind set, the performance measures of the SVM classifier were found to be: accuracy equal to 90.0%, sensitivity (TPR) equal to 80.0%, miss rate (FNR) equal to 20.0%, specificity (TNR) equal to 100.0%, precision (PPV) equal to 100.0% and AUC equal to 0.98.

One drawback of this study was the relatively small dataset, which can result in misclassifications, while the estimators may produce unstable and biased models, which can fail to generalize efficiently. However, the analysis of over-sampling SMOTE-generated datasets revealed that ML classifiers can also be effective for much bigger (up to 10-fold) datasets. In particular, the estimation of the performance measures of the optimized classifiers showed that all classifiers exhibited very good performances, yielding values above 0.84 for validation accuracy and above 0.84 for AUC. Additionally, SVM performed better according to the validation accuracy metric, while ensemble methods performed better according to the AUC metric. Considering accuracy as a more relevant metric for this supervised binary with balanced classes study, SVM was the selected method.

Therefore, as the results of this study demonstrate, the drawback of the small dataset size is surpassed by the dataset quality, namely the careful feature selection (e.g., CTCs), which provides significant information for the development of effective ML classifiers. In particular, our results indicate that flow cytometry, using the gating strategy described, can be a valuable tool for CTC enumeration with high sensitivity and specificity. In addition to the accuracy of the method, other advantages are also present. Additional markers can also be studied. Immunophenotyping CTCs, that is, the determination of the expression of markers related to steaminess or metastasis, could provide useful clinical information that can aid in cancer prognosis and/or treatment decisions. Additionally, using flow cytometry and sorting, CTCs can be isolated alive and cultured for downstream applications.

Overall, the results show that CTCs enumerated by flow cytometry can provide significant information, which when “fed” into ML algorithms can successfully discriminate between non-cancer/healthy and colorectal cancer patient subjects. Even though the results seem promising, more experiments have to take place in order to obtain larger datasets, while the exploitation of more sophisticated classification techniques is needed to verify and extend the results of this study. ML algorithms are not static products, and can continue to change and improve even once deployed, as new training data become available. However, these issues will be addressed in following studies. In conclusion, the results of this study are promising towards the development of a simple, fast and non-invasive screening method for cancer, using CTC enumeration by flow cytometry from blood samples and machine learning.

## Figures and Tables

**Table 1:** Validation accuracy of the optimized models, for datasets generated using SMOTE technique, using various parameters such as N = 1, 3, 10 and K = 5, 10, 20, 30.

	D1	D2	D3	D4	D5	D6
	N = 1	N = 1	N = 3	N = 3	N = 10	N = 10
	K = 5	K = 10	K = 10	K = 20	K = 20	K = 30
Trees	86.0	88.4	88.4	85.1	87.6	88.1
Discriminant	84.1	87.2	85.7	86.0	86.7	88.0
Logistic Regression	84.1	86.0	86.0	86.6	87.1	87.8
Naïve Bayes	84.1	86.6	85.7	86.0	86.9	88.1
SVM	86.0	89.0	89.6	87.2	87.3	88.0
KNN	85.4	87.8	89.6	85.7	84.4	87.9
Ensemble	86.0	88.4	88.4	87.2	86.9	88.2

**Table 2:** Estimated Area Under Curve (AUC) of the optimized models, for datasets generated using SMOTE technique, using various parameters such as N = 1, 3, 10 and K = 5, 10, 20, 30.

	D1	D2	D3	D4	D5	D6
	N = 1	N = 1	N = 3	N = 3	N = 10	N = 10
	K = 5	K = 10	K = 10	K = 20	K = 20	K = 30
Trees	0.89	0.88	0.92	0.88	0.94	0.86
Discriminant	0.89	0.88	0.91	0.92	0.94	0.93
Logistic Regression	0.89	0.88	0.91	0.92	0.94	0.95
Naïve Bayes	0.88	0.88	0.89	0.92	0.94	0.94
SVM	0.84	0.89	0.88	0.89	0.94	0.95
KNN	0.89	0.88	0.91	0.92	0.94	0.92
Ensemble	0.89	0.89	0.92	0.92	0.94	0.94

## Data Availability

The data are not publicly available due to containing personal information that could compromise participant privacy.
